# Oxidase enzymes as sustainable oxidation catalysts

**DOI:** 10.1098/rsos.211572

**Published:** 2022-01-12

**Authors:** Alice J. C. Wahart, Jessica Staniland, Gavin J. Miller, Sebastian C. Cosgrove

**Affiliations:** ^1^ Lennard-Jones Laboratories, School of Chemical and Physical Sciences, Keele University, Staffordshire, ST5 5BG, UK; ^2^ The Keele Centre for Glycoscience Research and Training, Keele University, Staffordshire, ST5 5BG, UK; ^3^ Croda Europe Ltd., Oak Road, Clough Road, Hull HU6 7PH, UK

**Keywords:** oxidases, biocatalysis, sustainable oxidation, alcohol oxidation, amine oxidation

## Abstract

Oxidation is one of the most important processes used by the chemical industry. However, many of the methods that are used pose significant sustainability and environmental issues. Biocatalytic oxidation offers an alternative to these methods, with a now significant enzymatic oxidation toolbox on offer to chemists. Oxidases are one of these options, and as they only depend on molecular oxygen as a terminal oxidant offer perfect atom economy alongside the selectivity benefits afforded by enzymes. This review will focus on examples of oxidase biocatalysts that have been used for the sustainable production of important molecules and highlight some important processes that have been significantly improved through the use of oxidases. It will also consider emerging classes of oxidases, and how they might fit in a future biorefinery approach for the sustainable production of important chemicals.

## 1. Introduction

Selective oxidation reactions are one of the most important transformations in the chemical sector. Typically, industrial oxidation chemistry is carried out using stoichiometric quantities of often undesirable reagents, such as chromium compounds. These harsh reagents often proceed with low levels of chemoselectivity, necessitating the need for protecting group chemistry and further reducing synthetic efficiency. The development of catalytic oxidation has increased the number of sustainable options that are available to chemists [1]. For example, earth abundant metals (Cu, Fe) in combination with TEMPO have been reported for the selective oxidation of primary alcohols [2].

**Scheme 1.**
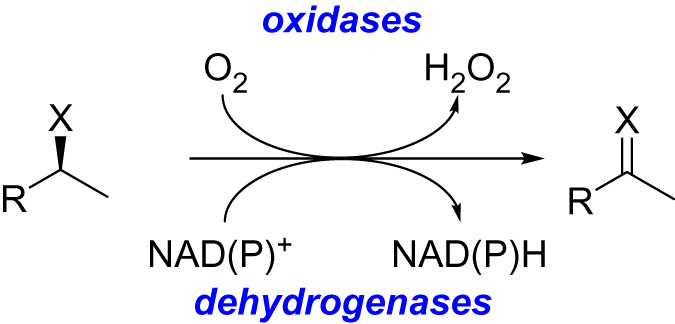
Biocatalytic approaches to oxidation are commonly undertaken with oxidases or dehydrogenases, X = OH, NH_2._

An alternative option for catalytic oxidation is the use of biocatalysis [3,4]. Enzymes are inherently sustainable, offering highly selective catalysts that work under mild conditions. More specifically, biocatalytic oxidation offers a green alternative to standard chemical methods. There are a host of biocatalytic options for enzymatic oxidation, including dehydrogenases, oxidases, monooxygenases and peroxygenases (scheme 1) [4]. Of these different enzyme classes, oxidases are particularly attractive from a green perspective. This is because they often solely depend on molecular oxygen as the terminal oxidant.

From a sustainability perspective, the exclusion of external stoichiometric oxidants is desirable. Being dependent exclusively on molecular oxygen as a co-substrate effectively reduces reagent input for oxidases to zero due to oxygen's natural abundance. It does present processing issues, however, primarily due to the low availability of oxygen in aqueous systems being limited to around 270 µM (8 mg L^−1^) [5]. This is sometimes below the kinetic requirement for engineered biocatalysts that depend on molecular oxygen, rendering their synthetic performance sub-optimal. To address this, one must design a process that maximizes the oxygen transfer rate to maintain the maximum aqueous concentration of oxygen, or be able to increase the amount of soluble oxygen in solution to the above ambient conditions (e.g. with a pressurized system). Understanding the kinetic requirements of oxidases is an important aspect of any oxygen-dependent bioprocess, providing data that informs better process design [6].

Most oxidases are not dependent on additional cofactors. Dehydrogenase enzymes depend on the addition of nicotinamide in *in vitro* systems. NAD(P)^+^/H is expensive, so is used catalytically with coupled recycling systems [7]. This can still be prohibitive on scale, with the use of sacrificial reagents to regenerate the NAD(P)^+^/H an additional waste source which decreases the overall sustainability of these bioprocesses. The flavin cofactors of many oxidases are co-expressed with and covalently bound to the protein, meaning there is no additional cofactor cost associated with their use in synthesis (table 1).

**Table 1.** Comparison of positive and negative aspects when using oxidase enzymes as sustainable oxidation catalysts.

oxidases	
pros	cons
no additional cofactor requirements	poor oxygen supply can limit enzyme performance
irreversible reactions	produce hydrogen peroxide as by-product
easy to engineer with coupled peroxide production in assay development	can suffer from substrate inhibition

This review will cover synthetic processes that have used oxidase enzymes, focusing on several important classes for synthesis, and the outlook for these enzymes as sustainable catalysts. In particular, it will discuss how different classes of oxidases have been used for bulk and speciality chemical syntheses, and how these different processes are enabling more efficient chemical transformations than previous chemical counterparts. It will also cover how different enzyme engineering approaches have been used to deliver toolboxes of starting points for further evolution and, in some cases, process-ready enzymes that can operate on an industrial scale as sustainable oxidation catalysts. There will also be a discussion of the future of oxidase biocatalysis, and how recently discovered enzymes (e.g. lytic polysaccharide monooxygenases (LPMOs)) could play a role in a full biorefinery approach to chemical production.

## 2. Discussion

### 2.1. Alcohol oxidases

Alcohol oxidation is one of the most important reactions in synthetic chemistry. While efficiently performed by a host of synthetic methods, many are incompatible with the green aims of society. The use of toxic metals such as chromium, and the frequent use of stoichiometric oxidants, renders them unsustainable. Enzymatic oxidation of alcohols has emerged as an attractive alternative, with a toolbox of different enzymes now available that are capable of oxidizing a large range of substrates [4].

Three different classes of enzyme that perform oxidation of alcohols include laccase, alcohol dehydrogenase/ketoreductase (ADH/KRED) and alcohol oxidases (AOx) (table 2) [4]. Laccases are Cu-containing enzymes that oxidize phenolic substrates, but require TEMPO or other electron mediators as a co-catalyst [8]. Alcohol dehydrogenases (ADHs) are broadly available enzymes which use NAD(P)H. In the context of this review, ADH and laccases will not be discussed.

**Table 2.** Classes of enzyme capable of alcohol oxidations.

classes of enzyme	cofactor
laccases	copper
alcohol dehydrogenase (ADH)	NAD(P)^+^/H
alcohol oxidases (AOx)	copper (CRO-OAx) or flavin (FAD-OAx)

AOx use oxygen (air), with coupled production of hydrogen peroxide as a by-product, to catalyse the oxidation of alcohols to the corresponding aldehydes or acids. AOx contain either a copper or flavin cofactor, converting primary and secondary alcohols to aldehydes and ketones, respectively.

### 2.2. Alcohol oxidases: copper radical alcohol oxidases

**Scheme 2.**
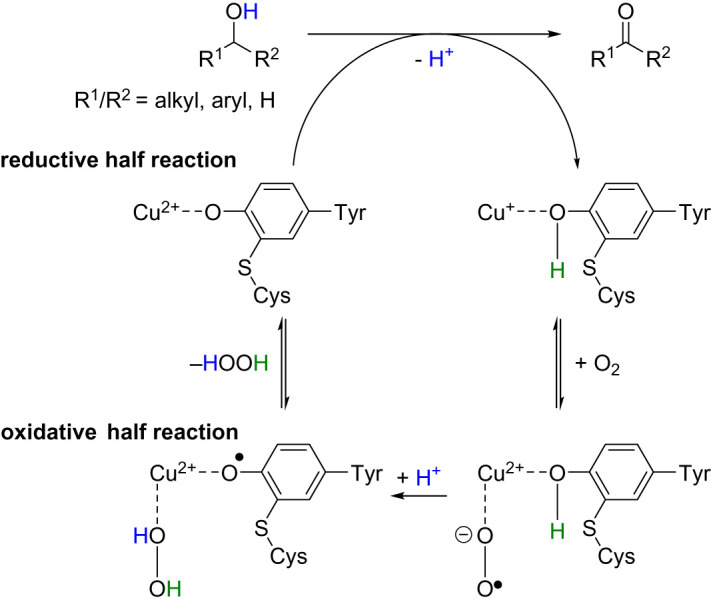
Catalytic cycle of copper-containing oxidases [10].

Regarding Cu-containing AOx, also named copper radical alcohol oxidases (CRO), the redox mechanism in galactose oxidase (GOase) was first described in 1959 by Cooper *et al.* [9]. Investigations revealed the mechanism likely proceeded via two consecutive single-electron transfer steps (scheme 2) [11,12].

**Scheme 3.**
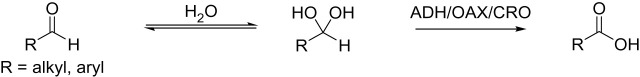
Aldehyde oxidation to carboxylic acid via gem-diol intermediate.

One advantage of CROs is that the oxidation usually stops at the aldehyde and oxidation to the acid is rarely observed. Aldehydes constitute an important class of molecule in flavours and fragrances (F&F), as described in a recent review [13]. The biotechnological production of aldehydes, such as fatty aldehydes, is becoming more common. For instance, Lafond and co-workers established the potential of a fungal AOx from *Colletotrichum graminicola* as a promising candidate for the production of several F&F aldehydes [14]. However, they also detected over-oxidation of 1-octanal, either via oxidation of the gem-diol intermediate, by a nonenzymatic mechanism, by the accessory enzymes (e.g. horseradish peroxidase (HRP) or catalase), or through a secondary oxidation with the AOx (scheme 3). They investigated the over-oxidation process by choosing two benzyl alcohol analogues bearing either an electron-withdrawing group (EWG, *p*-NO_2_) or an electron-donating group (EDG, *p*-OMe) to understand how this affected the hydration constant (*K*_H_). They showed the presence of an EWG increased over-oxidation to the acid (found to be 24% hydrate in aqueous solution), while an EDG produced only aldehyde (0% hydrate formation). They also detailed inhibition of the enzyme with long-chain fatty aldehyde hydrates, which allowed greater control in the oxidation of octanol.

### 2.3. Galactose oxidase

A frequently applied class of Cu-dependent oxidase is GOase. In Nature, it is responsible for the selective oxidation of galactosides at the C6 position. Several engineering campaigns have delivered GOase variants that possess much improved activity against a variety of useful substrates, including benzyl alcohols and the C6 hydroyxl group of glucosides. Due to the toolbox of variants which possess these broad activities, GOase has been referred to as a multifunctional catalyst [15].

One of the first synthetically useful GOase variants was engineered by Arnold and co-workers. They delivered a variant of the fungal GOase from *Fusarium graminearum*, termed M_1_, which had improved stability and expression in *E. coli*. [16]. This provided a platform for further engineering, with the groups of Arnold, Flitsch and Turner since adding significant contributions to this area [17–19]. As stated, these variants possess a diverse substrate scope, underlining GOase credentials as sustainable catalysts.

A useful report from Pedersen *et al.* discussed at length specific process requirements for GOase [20]. As well as outlining buffer requirements, the optimum amount of exogenous copper required, and demonstrating the long-term stability of GOase in solution, an important section stressed the need to design efficient oxygen supplies in GOase bioprocesses. The kinetic requirement of oxygen (*K*_MO_) for GOase was estimated to be above 5 mM in some instances, which is 20 times higher than the maximum oxygen concentration in aqueous buffers at atmospheric pressure. The authors described designing these types of processes as a trade-off between oxygen supply and enzymatic rate. This principle obviously counts for other oxidase enzymes as well.

An evolution study of the GOase M_1_ variant was described by Escalettes and Turner, with an aim to improve activity towards non-polar molecules (i.e. non-sugars) [18]. This was achieved, with new activity towards chiral secondary alcohols reported. A broad spectrum AOx for secondary alcohols was previously undisclosed, so this represented a significant step in the expansion of the synthetic capabilities of AOx. It was used for the kinetic resolution of a panel of secondary alcohols, with 13 of the substrates delivered with 99% *ee* after only 3 h. No details were disclosed as to scale or concentrations, but the final M_3-5_ variant was said to be 2000-fold less active towards d-galactose, which represented a significant alteration of the biocatalyst function.

**Scheme 4.**
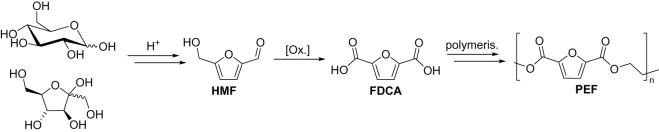
PEF production from sugars via HMF and FDCA.

There have been several subsequent studies that highlight the potential for GOase as a catalyst for sustainable manufacture. Perhaps the most significant is in the oxidation of 5-hydroxymethylfurfural (HMF), a key intermediate in the production of bio-based plastic alternatives [21]. It can be formed by the dehydration of glucose or fructose (cellulose feedstock) and then oxidized to form 2,5-furandicarbxoylic acid (FDCA). FDCA is the key raw material for polyethylene furandicarboxylate (PEF), developed by Avantium [22], which is a bio-based plastic alternative to fossil-based polyethylene terephthalate (PET). PEF production has been shown to have the potential to reduce energy use by 40–50% and greenhouse gas emissions by 45–55% in comparison to conventional PET plastic production [23,24]. Furthermore, PEF bottles have superior properties to PET bottles including improved gas permeability and a higher melting point. However, the vast majority of FDCA syntheses from HMF involve harsh conditions, so biocatalytic alternatives are attractive and being actively sought (scheme 4).

There have been several reports of engineered biocatalysts that are capable of producing oxidation products of HMF. More specifically, however, there are a number of examples of GOase variants being used for HMF oxidation. In 2015, McKenna *et al.* [25] demonstrated that GOase M_3-5_ was highly effective in the oxidation of HMF to the dialdehyde 2,5-diformylfuran (DFF) and could be coupled with a second oxidase enzyme to facilitate the full oxidation to FDCA. The substrate concentrations were limiting in this cascade with a maximum of 20 mM HMF being demonstrated, which is clearly not compatible with bulk chemical production.

In a subsequent, recent report, Birmingham *et al*. [26] described new GOase variants that showed much improved performance in the selective oxidation of HMF to DFF. After engineering several new mutants, the authors postulated that a mutant with activity at lower oxygen concentration would have improved overall performance (i.e. lower *K*_MO_). To find such variants, the screening assay was performed in a glove box with a low oxygen concentration (0.2% v/v). They found a mutation of the F290 residue in the M_3-5_ variant to a tryptophan (F290W) significantly improved oxygen binding. Interestingly, the wild-type GOase had a W290 residue which was changed to phenylalanine during the M_3-5_ evolution study. The F290W mutation was found in two of the mutants under low oxygen concentration and resulted in an increase of the *k*_cat,app_ for HMF from 123.9 s^−1^ for M_3-5_, to 200.5 s^−1^ for the M_6-A_ variant. The use of a tube-in-tube reactor enabled exact determination of the *K*_MO_ for the different variants [27]. It was calculated that the M_3-5_ variant had a *K*_MO_ of 1.39 mM, well above the ambient availability, with the M_6-A_ variant shown to have a much lower value of 0.15 mM. Combination of several key residues identified from additional rounds of evolution delivered final variants, termed M_7_, that could convert semi-pure samples of HMF at 100 g L^−1^ substrate loading (approx. 790 mM), with the best mutant (M_7-2A_) affording a biocatalyst productivity of 1500 g_DFF_ g_enzyme_^−1^ and 96% conversion after only 6 h (analytical scale). Industrial partners BASF scaled this variant to 1.44 L scale, producing DFF in a 92% isolated yield at 31.5 g L^−1^ (250 mM) HMF loading (table 3). This clearly shows the potential for GOase variants to use HMF as a bio-based feedstock; however, work is still required to realize a fully scalable biocatalytic synthesis of FDCA.

**Table 3.** Comparison of different GOase variants for HMF oxidation in Birmingham *et al.* [26] conditions: CuSO_4_ (50 μM), HRP (4 U ml^−1^), NaPi buffer (100 mM, pH 7.4), 0.9 vvm air, 20°C, 6 h.

GOase variant	[HMF] (g l^−1^)	[GOase CFE] (g l^−1^)	[catalase] (U ml^−1^)	scale	conv./yield	productivity
M_3-5_	50	0.9	48	200 mL	24%/−	232 g_DFF_ g_biocat_^−1^
M_6-A_	50	1.57	58	200 mL	37%/−	333 g_DFF_ g_biocat_^−1^
M_7-2A_	50	0.96	305	200 mL	59%/−	516 g_DFF_ g_biocat_^−1^
M_7-2A_^a^	31.5	0.05^b^	n/a	1.44 L	−/92	570 g_DFF_ g_biocat_^−1^

^a^Performed separately by BASF.

^b^Purified enzyme.

**Scheme 5.**
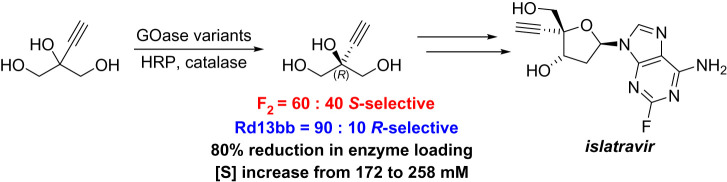
Engineered GOase variant for oxidative desymmetrization of glycerol intermediate for islatravir synthesis.

A different example of an engineered GOase was recently disclosed by Huffman *et al.* [28]. The study described the design of a full biocatalytic route to the nucleoside analogue islatravir, an investigational HIV treatment. A nine-enzyme cascade, including five that were engineered, transformed a glycerol derivative into the final product. The GOase F_2_ variant provided a starting point for the desymmetrization of 2-ethylnyglycerol. The stereoselectivity of the F_2_ variant was incorrect, favouring the *S*-aldehyde in a ratio of 60 : 40. A 12-round evolution campaign improved enantioselectivity and overall activity, eventually delivering a process suitable biocatalyst (GOase Rd13bb) that afforded the correct aldehyde in 90 : 10 ratio (scheme 5). The remaining (*S*)-enantiomer was also further over-oxidized to the acid by the GOase if the reaction was left for longer, which did bring the *ee* up to 99% but also resulted in a slightly reduced overall yield (approx. 70%).

With so many sequential reactions occurring in a single vessel, reversible biocatalytic reactions (such as hydrogenase) could be hard to balance. As oxidase reactions are irreversible, this highlights the benefit of employing them in instances such as that above.

### 2.4. Alcohol oxidases: flavin dependent

**Scheme 6.**
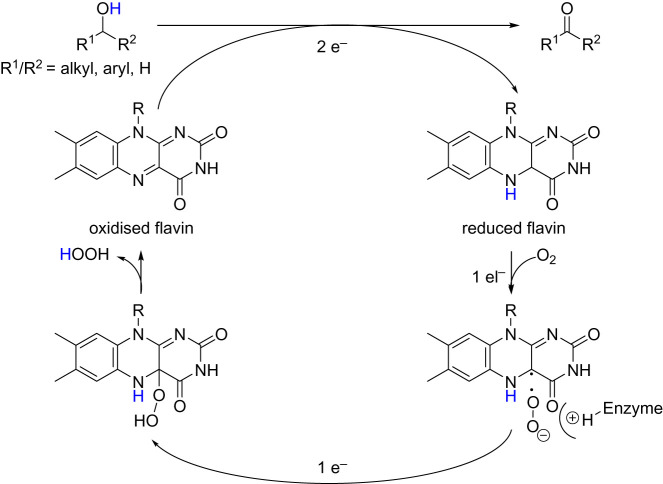
Catalytic cycle of flavin-containing AOx [10].

Flavin-AOx are multimeric enzymes, in which the flavin cofactor can be either covalently bound (vanillyl-alcohol oxidase flavoprotein family) or with a flavin-binding domain (glucose–methanol–choline flavoprotein family) [29]. There are two possible forms of flavin, either flavin adenine dinucleotide (FAD) or flavin mononucleotide (FMN). Oxidation using AOx containing flavin proceeds via two half reactions, where the alcohol is first oxidized by a two-electron transfer yielding reduced flavin. The oxidized flavin is regenerated by a stepwise single-electron transfer. Oxygen acts as single-electron acceptor and forms superoxide (O_2_^−•^), stabilized by a positively charged histidine residue. Another single-electron transfer yields a covalent hydroperoxy flavin intermediate, which eliminates hydrogen peroxide and reforms oxidized flavin (scheme 6) [10,30].

**Scheme 7.**
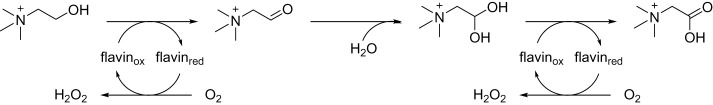
Two-step oxidation of choline by choline oxidase yielding betaine.

The oxidation of primary alcohols catalysed by flavoprotein oxidases does not necessarily stop at the aldehyde stage and may proceed to the corresponding carboxylic acid. This second oxidation occurs via the aldehyde hydrate (*gem*-diol, see scheme 3), as shown with choline oxidase (E.C. 1.1.3.17, scheme 7) [31]. Not all FAD-AOx oxidize to the carboxylic acid as demonstrated by Pickl and co-workers [10].

**Scheme 8.**
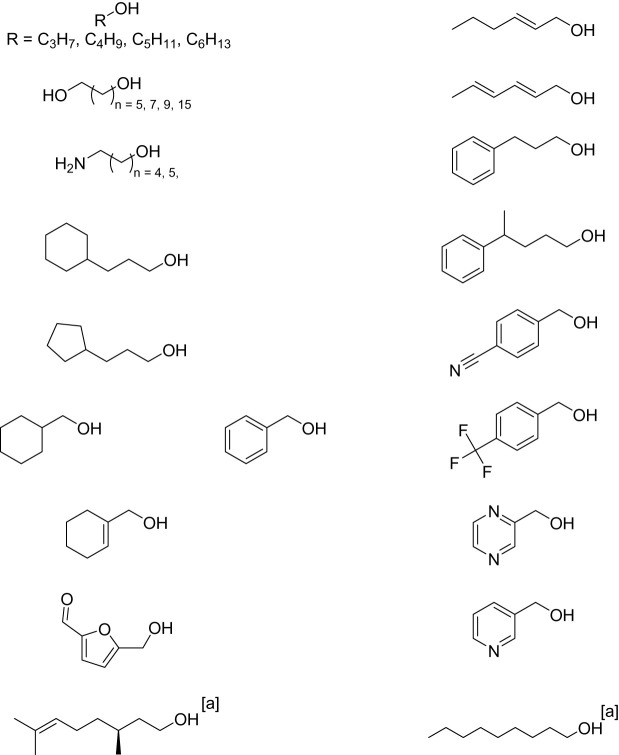
Reported substrate scope of AcO6 [a] *in vivo.*

Tuner and co-workers focused on using structure-guided mutagenesis to evolve the choline oxidase from *Arthrobacter cholorphenoculicus* (AcCO) in order to generate a mutant with a wide substrate scope for the oxidation of primary alcohols [32]. Using the crystal structure of AcCO allowed structure-guided evolution; the active site and access channel were identified as key areas for mutagenesis to alter the substrate scope of the enzyme. The most active variant, termed AcCO6, had six mutations, including two which were remote from the active site. The AcCO6 mutant had higher thermotolerance and better conversion in organic solvents than the wild-type AcCO. Multistep synthesis using AcCO6 has been reported in a sequential amination cascade with aminating enzymes, in both batch [33] and more recently in continuous flow using a specially designed flow reactor for the enhancement of oxygen-dependent biocatalysts [5,34]. Recently, AcCO6 has also been studied as a catalyst within an *in vivo* system for the first time [35]. In this report, a LuxAB biosensor system was implemented in *E. coli* to monitor the enzymatic production of aldehydes from primary alcohols and carboxylic acid substrates. This coupled system was used to identify the *in vivo* production of a range of aliphatic and aromatic aldehydes for the first time, and was even used to oxidize the aldehydes to their respective acids in some instances, allowing a two-step biocatalytic oxidation cascade to occur. A summary of some of the different substrates that have been oxidized using the AcCO6 variant is shown below (scheme 8).

The removal of glycerol as a by-product of biodiesel production is an important line of investigation to reduce environmental impact. Some biocatalytic methods have been investigated using AOx. A noteworthy example was the comprehensive structural and biochemical characterization of an AOx from the white-rot basidiomycete *Phanerochaete chrysosporium* (PcAOx) [36]. Methanol, ethanol and propan-1-ol seemed to be good substrates for the wt PcAOx, but glycerol was a poor substrate, even when tested against higher substrate concentrations (1–4 M). Mutation of a few key residues (F101 and M103) converted the wild-type into a better glycerol oxidase. One of the final mutants (F101S) had its crystal structure resolved to reveal an increase of the active site cavity volume from 37 Å^3^ to 127 Å^3^. The activity towards glycerol was still quite low (*K*_M_ = 580 mM, *k*_cat_ = 3 s^−1^); however, this presented a much improved variant than the wild-type. This biocatalyst could provide a platform to reduce the impact of biodiesel production.

The Fraaije Group recently demonstrated that the PcAOx F101S variant was capable of the double, and sometimes triple, oxidation of a range of aliphatic diols (table 4) [37]. The cyclization of the hydroxy aldehyde intermediates delivered lactol intermediates which, depending on the stability of the lactol, was enzymatically converted to either the hydroxy acid or the lactone. The authors determined steady-state kinetic parameters of several of the substrates that were screened. Interestingly, the catalytic efficiency (*k*_cat_/*K*_M_) value for heptanal (750) was around 4.5-fold higher than heptanol (187). This obviously shows that control using these enzymes is difficult, with the *K*_M_ value for the aldehyde being over 10 times lower than the alcohol. Another aspect highlighted was the selective oxidation of secondary alcohols, a substrate class that is very limited with respect to FAD-AOx. The activity was low (15% conversion of 1-phenylethanol in 48 h) but showed practical oxidation could be possible with further evolution of this enzyme.

**Table 4.** Diols and other alcohols oxidized using the PcOAX mutant *Substrate (20 mM), PcAOx (40 *μ*M), KPi buffer (100 mM, pH 7.5), 48 h, 35°C.

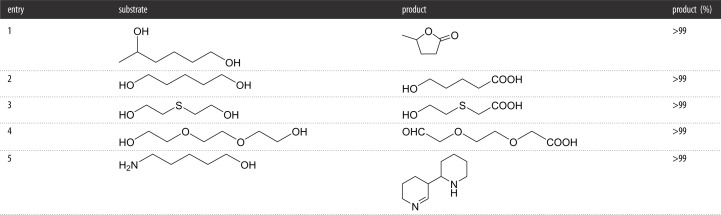

The authors further demonstrated this activity towards secondary alcohols with the kinetic resolution of several chiral alcohols (table 5) [38]. The *ee* was perfect for a couple of substrates, but low for the propargyl substrate. As with many oxidases, the substrate concentration was limiting, as well as the reaction time (20 mM, 24 h), and the paper did not disclose a significant substrate scope.

**Table 5.** Kinetic resolution of secondary alcohol using PcOAX as biocatalyst.

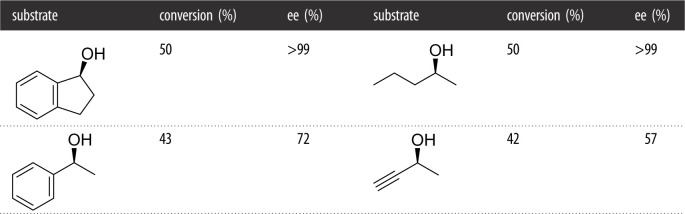

**Figure 1.**
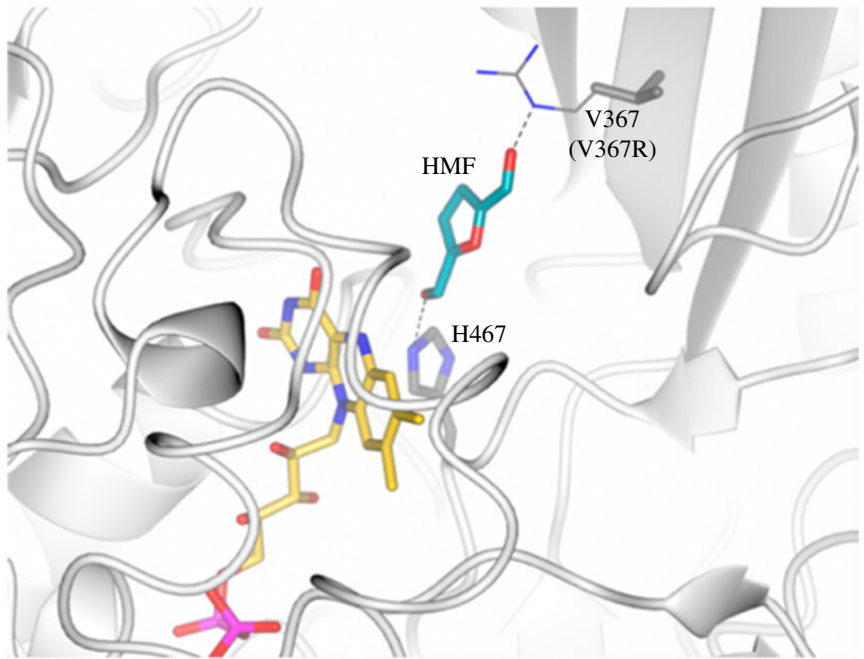
Proposed model for the binding of 5-hydroxymethylfurfural substrate in HMFO active site. Reprinted with permission from Mattevi *et al.* [39]. Copyright 2015 American Chemical Society.

5-Hydroxymethylfurfural oxidase (HMFO) is a flavin-dependent enzyme which catalyses the conversion of sugar-based HMF to the bioplastic precursor FDCA (see above). In 2015, Mattevi and co-workers [39] solved the HMFO crystal structure, which permitted an evolution campaign to improve its activity. The wild-type HMFO can oxidize primary alcohols only. For this reason, mutant enzymes were designed which could convert both HMF and secondary alcohols ((*S*)-1-phenylethanol) to the respective carbonyls. Mutagenesis experiments revealed two key residues in the active site, namely V367 and W466 (figure 1). The HMFO W466F and W466A variants allowed for an enlarged cleft in the active site, and this expanded pocket resulted in activity towards bulkier secondary alcohols. They showed low activity towards (*S*)-1-phenylethanol, with a *k*_cat_ of 0.01 s^−1^ for both variants and *K*_M_ values for 20 mM and 98 mM for the W466A and W466F mutants, respectively. They had perfect stereoselectivity though, with no observed activity towards the (*R*)-enantiomer. This activity towards bulkier substrates inspired the design of the final HMFO, as secondary alcohols are iso-structural to *gem*-diols. Their mutant HMFO V367R resulted in better HMF to FDCA conversion (approx. threefold higher) than the wt, with a crystal structure demonstrating the close proximity of the V367 residue to the hydroxymethyl arm of the HMF substrate. A combination of the mutations gave the V367R-W466F double mutant, which had a 1000-fold higher catalytic efficiency (*k*_cat_/*K*_M_, 2.2 s^−1^ mM^−1^) value than the wild-type HMFO (0.0005 s^−1^ mM^−1^) towards HMF.

The HMFO oxidase was capable of catalysing the full oxidation to FDCA, which is obviously an improvement over previous reports for this challenging substrate. Nevertheless, the reaction was not intensified and no scale up reaction was reported in this instance. Further details disclosing how this enzyme performs on a preparative scale are needed before comparison can be made with other HMF oxidation bioprocesses.

### 2.5. Amine oxidases

Amine oxidase (AmOX) is a class of enzymes that mediate the selective oxidation of amines to the respective imines [40]. Imines are important functional groups for organic synthesis, allowing the formation of (chiral) α-substituted amines. Selective amine oxidation is one of the most challenging synthetic transformations, highlighting the importance of this biocatalyst for the development of sustainable methods for the production of imines.

As with AOx, there are two classes of AmOX: copper dependent and flavin dependent. The flavin-dependent class has been used more widely in synthesis, with this attributed to the mechanism: the flavin cofactor does not covalently bind the imine, releasing it upon formation. The copper-dependent AmOX retains the imine, which must be hydrolysed before it can be released into solution, limiting the application of this class in synthesis [41]. The mechanism proceeds broadly in the same manner as the AOx, which has permitted evolution through coupling to peroxide detection assays as well.

### 2.6. Monoamine oxidase from *Aspergillus niger*

**Scheme 9.**
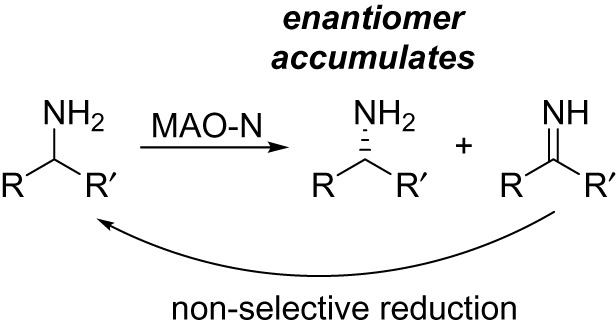
Biocatalytic deracemization process using MAO-N.

The monoamine oxidase from *Aspergillus niger* (MAO-N) is a flavin-dependent AmOX. It is similar in structure to the known mammalian MAO-A and MAO-B, with the wild-type first reported by Schilling and Lerch in 1995 [42]. The substrate scope of the wild-type was limited to simple alkylamines such as butyl- and amylamine, however, considerable engineering campaigns, primarily driven by the Turner group, have now delivered a range of MAO-N variants with a broad substrate scope [43]. They exploited the perfect (*S*)-stereoselectivity of MAO-N to develop a chemoenzymatic dynamic kinetic resolution, or deracemization, protocol for racemic chiral amines. The enzyme selectively oxidizes one enantiomer, giving a mixture of one enantiomer and the imine. This is coupled to a non-selective reduction of the imine with a reducing agent, which then undergoes the same cycle gradually building up the single enantiomer of which the enzyme is not selective for (scheme 9).

The first variant of MAO-N was evolved for activity towards methylbenzylamine (MBA) [44]. The evolution was performed using a colourimetric HRP assay that detected the hydrogen peroxide by-product of the reaction. The HRP enabled oxidation of a dye, with any red *E. coli* colonies that appeared on the plate indicating activity towards the substrate. The final mutant contained a single mutation (N336S) and was able to convert a racemic mixture of MBA to the *R*-enantiomer in 93% ee.

Since this first report, there have been multiple studies published, and there are now three MAO-N variants with a broad substrate scope that are used, termed D5, D9 and D11 [41,43]. They were evolved to demonstrate an increased substrate scope versus the wild-type and earlier variants, therefore improving their synthetic utility, with some key examples described below.

**Scheme 10.**
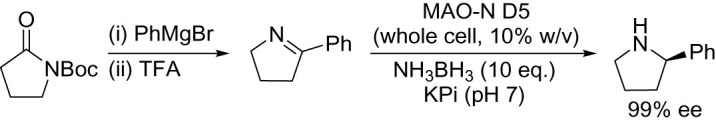
Three-step synthesis of (*R*)-2-phenylpyrrolidine using a chemoenzymatic deracemization.

The D5 variant, which was the first of these three broadly active MAO-N variants to be reported, contained five single-point mutations (I246M/N336S/M348K/T384N/D385S) versus the wild-type [45]. It was noted that it had particularly high activity towards cyclic amines, both secondary and tertiary. The authors detailed the synthesis of 2-phenylpyrrolidine, using the MAO-N D5 variant to deracemize the racemic cyclic amine (Formed *in situ*, scheme 10).

Soon after this report, Grogan, Brzozowski and co-workers were able to obtain a crystal structure of the D5 variant, which allowed a greater understanding of the active site, and helped directed evolution efforts for the additional variants [46]. The mutations were not all in the active site, with two of the mutations at positions T384 and D385 found to cause a change in the protein structure which resulted in an increase in the size of the pocket. This allowed larger substrates to enter the active site, which in turn increased the substrate scope of the enzyme.

**Scheme 11.**
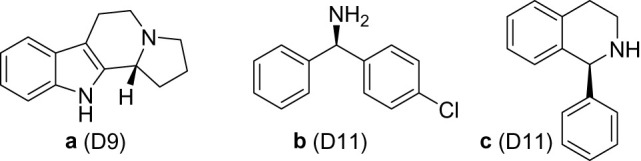
Different molecules synthesized with MAO-N variants.

As stated, the engineering of MAO-N has delivered a toolbox of variants. The D9 and D11 variants contained additional mutations that vastly increased the substrate scope to encompass more complex molecules such as (*R*)-harmicine **a**, and precursors to levocetirizine **b** and solifenacin **c** (scheme 11).

**Scheme 12.**
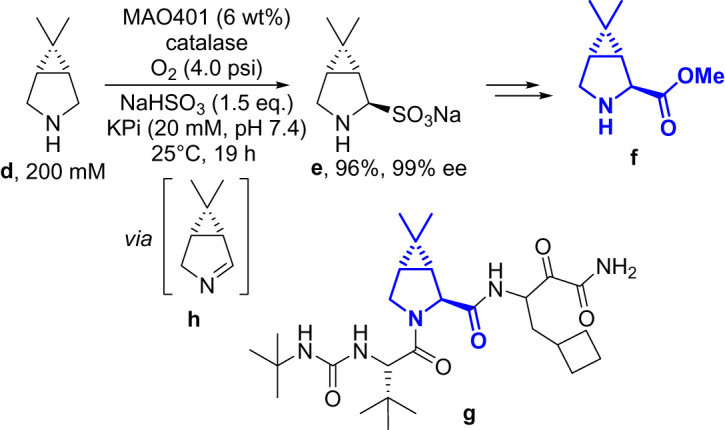
Boceprevir and the fragment synthesized using an engineered AmOX.

The wild-type MAO-N also provided a starting point for researchers from Merck in their synthesis of a pyrrolidine fragment **f** used in the manufacture of protease inhibitor boceprevir **g** [47]. The previous medicinal chemistry route had employed a standard chemical resolution procedure to obtain the enantiopure pyrrolidine **f**. The authors stated that the efficiency was not particularly high, with 50% of the material discarded after the resolution. They described an evolution campaign that delivered a highly efficient final variant (MAO401) that was used in commercial production of **e** (scheme 12). They used an AmOX-mediated oxidation of *meso*-pyrrolidine **d** to afford the imine **h**, which they found inhibited the enzyme and prevented conversion above approximately 45%. An *in situ* capture of the imine using bisulfite generated **e** which was highly water soluble and did not have a detrimental effect on enzyme activity.

Interestingly, the authors compared some of the metrics of the resolution method and the MAO route, and found the biocatalytic route they designed significantly improved both performance and the overall sustainability. There was roughly a 60% reduction in raw materials used, total amount of water used and the E-factor. This was a clear demonstration of the sustainability benefits switching to a biocatalytic route can have for a manufacturing process and in particular the advantages of using oxidases in a process environment.

Additional (*S*)-selective AmOX have also been reported, including engineered cyclohexylamine oxidase variants which were evolved for activity towards tetrahydroquinolines [48–50].

### 2.7. 6-hydroxy-d-nicotine oxidase (6-HDNO)

While the AmOX variants derived from *Aspergillus niger* have seen widespread application, they have only ever been reported as (*S*)-selective. The Turner group addressed this through development of an (*R*)-selective AmOX, based on 6-hydroxy-d-nicotine oxidase (6-HDNO) [51]. The wild-type enzyme selectively catalyses the oxidation of the 2-position of the pyrrolidine moiety in the hydroxylated d-nictoine during metabolism. Two key active site residues were identified (E350, E352) to be involved in catalysis, so site-saturation libraries were constructed at these positions. This revealed an active variant, namely E350L/E352D, as the best, which also possessed a broad substrate scope with activity towards 19 of the 34 substrates that were screened (the wild-type was only active towards eight of the same panel). Asano and co-workers also engineered an (*R*)-selective amino acid oxidase from *porcine kidney* (*Pk*DAO). In Nature, this enzyme catalyses the oxidative deamination of amino acids, but a couple of mutations altered the specificity to diminish amino acid activity and increase its capacity to selectively oxidize (*R*)-alkyl amines [52].

**Scheme 13.**
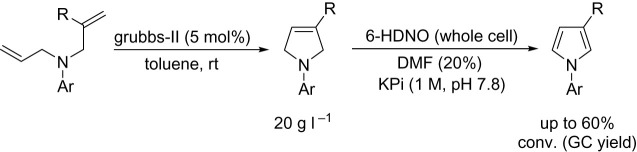
Two-stage chemoenzymatic pyrrole synthesis with Grubbs-II catalyst and 6-HDNO.

There have been several applications of 6-HDNO in (chemo)enzymatic cascade reactions. The original report detailed the use of the biocatalytic deracemization reaction (see scheme 5), but a later report showed the reducing agent could be replaced with an imine reductase to allow for a fully biocatalytic deracemization [53]. Additionally, Castagnolo and co-workers have presented several examples of AmOx (both MAO-N and 6-HDNO) in combination with transition metal catalysts for the synthesis of heterocycles. Specifically, they described a sequential Grubbs/6-HDNO sequence whereby bis-allylamines could be converted to the respective pyrroles (scheme 13). The oxidation reactions were run with a 4 : 1 buffer/DMF mixture, which permitted concentrations of 20 g L^−1^ to be achieved, impressive for this class of enzyme.

Approaches that can combine the power of both synthetic and biocatalysts are an attractive way to think about chemical synthesis. Designing synthetic routes that use both types of catalysts will ultimately prove to be more efficient, and therefore more sustainable when compared to individual routes. For example, prior reports using a similar approach (Grubbs/oxidative aromatization) relied on acid-mediated elimination. Replacing the latter step with enzymatic approaches is more compatible with future needs for sustainable syntheses.

### 2.8. Oxidases in multi-enzyme cascades

The realization that biocatalysts can operate with near-perfect chemoselectivity has seen them increasingly applied in multi-enzyme cascades [54–56]. There have been several reports of oxidase enzymes being combined with other biocatalysts to build molecular complexity. Oxidases lend themselves well to cascade reactions as they typically generate reactive species (carbonyls, imines, etc.) and due to their irreversible nature can provide a driving force in a biocatalytic reaction, contrary to some combinations of dehydrogenases. These features were exploited by Ramsden *et al*. when they combined the choline oxidase AcCO6 variant with a reductive aminase (RedAm) to convert a range of alcohols to their respective *N*-substituted amines [33]. Previous iterations of this cascade had co-deployed ADHs in the oxidative direction with aminating enzymes such as RedAms or amine dehydrogenases, which allowed a closed-loop recycling of the nicotinamide. These cascades could be affected by the thermodynamic stability of the products as both reactions were reversible. This was overcome with the AcCO6 variant as the oxidation was irreversible. This cascade was also transferred into continuous flow, with a flow reactor that supplied above ambient *in situ* soluble oxygen, which enhanced the rate of the enzyme [34]. For example, the batch reaction was limited to 25 mM substrate concentration and took 24 h to go to completion on a preparative scale. When transferred to flow, the substrate concentration was increased to 60 mM and proceeded to go to full completion in only 11 min.

**Scheme 14.**
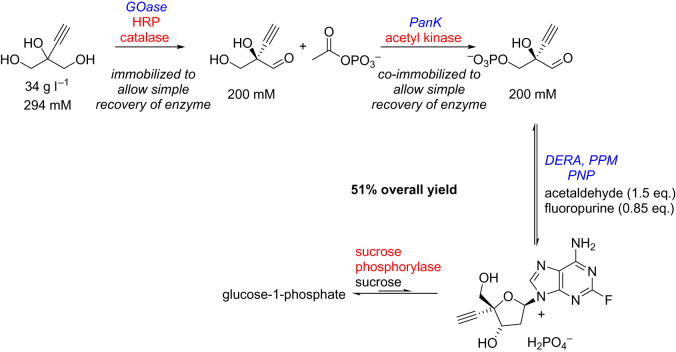
Biocatalytic cascade synthesis of islatravir. Enzymes in blue were engineered in the study [28]. GOase = galactose oxidase, HRP = horseradish peroxidase, PanK = pantothenate kinase, DERA = deoxyribose 5-phosphate aldolase, PPM = phosphopentamutase, PNP = purine nucleoside phosphorylase.

A pioneering example, discussed briefly already, was the nine-enzyme cascade which included an engineered GOase for the total biocatalytic synthesis of experimental HIV drug islatravir (scheme 14) [28]. The GOase was responsible for setting the stereochemistry at the C5 position of the ribose in the final product. Overall, the cascade proceeded with an isolated yield of 51% from the glycerol derivative. While no sustainability metrics were calculated within this manuscript, the authors noted the prior synthetic route was more than twice as long, with a specific comment on the significant improvement in atom economy using this biocatalytic route.

The development of new enzymes will open opportunities for novel cascades to be developed.

### 2.9. Lytic polysaccharide monooxygenases

As the importance of a sustainable bioeconomy emerges, so do challenges in providing sources of biofuel to replace traditional fossil fuels. So-called first-generation biofuels are derived from edible materials, such as starch, but have limitations due to their relative abundance. A second generation of biofuel derives from non-edible plant biomass and offers a solution here as this is the most abundant biopolymer on the planet. Such material is composed of lignocellulose (cellulose, hemi-cellulose and lignin) and for several years the concept of creating biorefineries to break down these polysaccharides into their constituent building blocks for use as biofuels or ‘green’ chemical building blocks has edged toward reality [57].

Deconstruction of complex carbohydrate biopolymers is however a limitation in utilization of biomass as a biofuel. The complexes present as heterogenous composites, resistant to degradation and are inherently stabilized by hydrogen-bonding interactions and stacking of individual polymer chains. Traditional methods using chemical approaches to deconstruct and functionalize cellulosic-based materials now synergize with enzymatic approaches.

**Figure 2.**
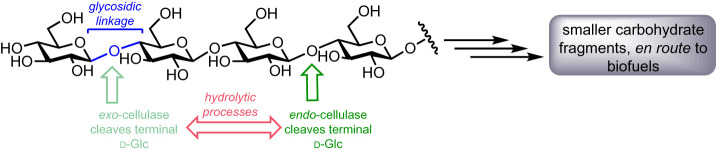
Structure of cellulose with glycosidic cleavage points for canonical *exo*- and *endo-*cellulase activity.

Within this biorefinery concept, carbohydrate-active enzymes (CAZys) are the key catalysts for efficiently degrading polysaccharide biomass. For example, several classes of hydrolytic enzyme can degrade cellulose, acting both internally and externally upon the polysaccharide chain (figure 2). Despite significant effort towards developing such degradative capabilities, the realization of dominant industrial-scale applications is yet to emerge.

**Figure 3.**
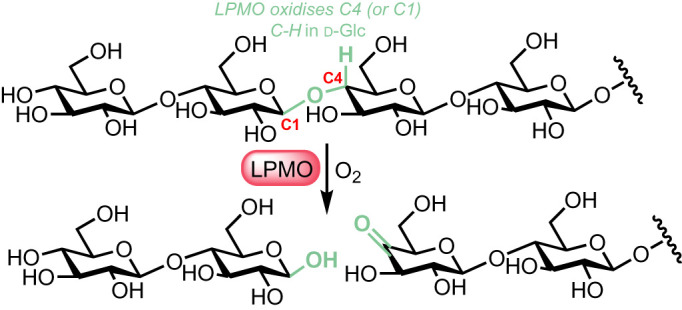
Oxidative cleavage of glycosidic linkages by LPMOS (C4 cleavage shown).

However, a significant breakthrough towards realizing this arrived in 2010 with the identification of a different class of enzyme, able to cleave polysaccharide inter-glycosidic linkages using an *oxidative process*. So termed LPMOs (an Auxiliary Activity CAZy classification) are oxygen dependant metalloenzymes and while first characterized cleaving chitin polysaccharides [58], their activity upon cellulose was soon demonstrated and X-Ray structures solved to elucidate mechanistic understanding [59–62]. These enzymes break glycosidic linkages through an oxidation at the C1 or C4 positions (C–H to C–OH), followed by hemi-acetal deconstruction to ketone and hemi-acetal products (figure 3).

**Figure 4.**
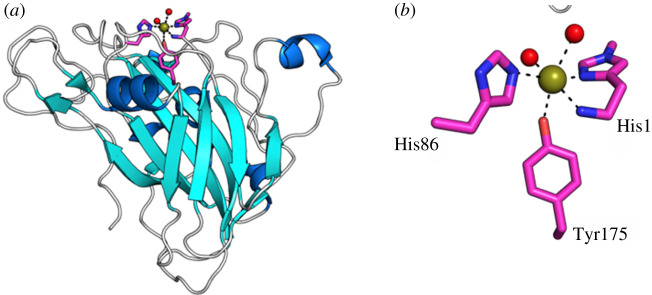
(*a*) Three-dimensional structure of a typical LPMO and (*b*) histidine brace complex coordinating active site Cu^II^ (adapted with permission from reference [63]).

Within the LPMO active site, a copper (II) metallo-component is coordinated by a terminal *N*-methylated histidine residue, a tyrosine and an internal histidine, now commonly termed as the ‘histidine brace’ (figure 4); complexities surrounding a complete mechanistic understanding of this catalytic mechanism mean it is still an area of intense research [64,65].

Beyond mechanistic understanding of LPMO structure-to-function relationships [66], research into their full operational use and characterization is also an active area of research [63,67]; particularly, aspects surrounding recombinant production of active LPMOs, basic and accurate characterization of activity upon polysaccharide substrates, and reaction kinetics. This is alongside consideration of practical use issues, surrounding side reactions, self-inactivation and the fuelling of such processes (H_2_O_2_ versus O_2_).

In many respects, the resolution of these complexities within this exciting class of CAZy is needed before a complete transition into real-world biorefinery applications can be enacted. Nonetheless, there are examples of relevant patents that pre-date the recent reclassification of LPMOs within the CAZy database [68], and LPMOs are a component of Cellic CTec enzyme products. LPMOs add significant new strength to biorefinery-oriented polysaccharide degradation cocktails, boosting the activity of canonical cellulose hydrolases and thus reducing overall enzyme loading for such processes [68–70].

The field of LPMO research and application is burgeoning. As further advances in their characterization and mode of action are made, alongside the identification of new enzymes [71], their utilization as oxidative biocatalysts will no doubt emerge onto an industrial platform.

### 2.10. Outlook for oxidases as sustainable catalysts

The chemical industry has a particularly challenging task when it comes to decarbonization due to the high temperatures that are used in many processes. Finding ways to improve the sustainability of these processes is a priority for many companies. Biocatalysis offers a potential solution for a range of chemical reactions. With low operating temperatures, targeted synthesis and biodegradability, enzymes offer a more sustainable route to existing products and allow access to new product ranges. For some chemical industry sectors, reactions such as oxidations are avoided due to the hazardous nature of the traditional catalysts. Oxidases may allow access to novel compounds with functional benefits that are not currently deemed to be achievable. The benefit of using a biodegradable oxidation catalyst that only requires oxygen as a terminal oxidant cannot be understated. Challenges associated with scale up and enzyme stability still limit widespread application on industrial scales, but with advancements in protein engineering coupled to the development of new reactor technologies, solutions to these problems are being presented more frequently.

More generally, biocatalysis lends itself to the lights out manufacturing philosophy, the idea of factories that are entirely automated and operate with no human interaction, therefore not needing lights. This is a concept the chemical industry has yet to adopt widely if at all. The lower risk associated with the use of enzymes in manufacturing (lower temperatures, non-toxic reagents) would de-risk the movement towards automated chemical manufacture, unlike more traditional chemistry which must be more closely monitored. This philosophy could play an even more important role for industrial oxidations moving forward and present a perfect opportunity to adopt oxidases as sustainable catalysts across the chemical sector.

## Data accessibility

No new data is associated with this article.
